# Crystal structure of *N*-(quinolin-6-yl)hydroxyl­amine

**DOI:** 10.1107/S160053681402193X

**Published:** 2014-10-11

**Authors:** Anuruddha Rajapakse, Roman Hillebrand, Sarah M. Lewis, Zachary D. Parsons, Charles L. Barnes, Kent S. Gates

**Affiliations:** a 125 Chemistry Bldg, University of Missouri Columbia, MO 65211, USA

**Keywords:** crystal structure, *N*-aryl­hydroxyl­amine, hydroxyl­amine, *N*-(quinolin-6-yl)hydroxyl­amine, quinoline

## Abstract

The title compound, C_9_H_8_N_2_O, crystallized with four independent mol­ecules in the asymmetric unit. The four mol­ecules are linked *via* one O—H⋯N and two N—H⋯N hydrogen bonds, forming a tetra­mer-like unit. In the crystal, mol­ecules are further linked by O—H⋯N and N—H⋯O hydrogen bonds forming layers parallel to (001). These layers are linked *via* C—H⋯O hydrogen bonds and a number of weak C—H⋯π inter­actions, forming a three-dimensional structure. The crystal was refined as a non-merohedral twin with a minor twin component of 0.319.

## Abstract 2

The title compound crystallized with four independent mol­ecules in the asymmetric unit. They are linked *via* two N—H⋯O and one O—H⋯N hydrogen bond, forming a tetra­mer-like unit.

## Chemical context   

*N*-Aryl­hydroxyl­amines can be generated in chemical, biochemical and biological systems either by reduction of nitro­aromatic compounds or oxidation of aryl­amines. Inter­estingly, few aryl hydroxyl­amines have been crystallographically characterized. In part, this may be due to the instability of these compounds. For example, *N*-aryl­hydroxyl­amines can undergo spontaneous oxidation to generate the nitroso derivatives (Rubin *et al.*, 1987 ▶; Veggi *et al.*, 2008 ▶). These compounds, in turn, condense with the unreacted hydroxyl­amine to yield the az­oxy derivatives (Pizzolatti & Yunes, 1990 ▶; Agrawal & Tratnyek, 1996 ▶). They are also of particular importance as inter­mediates in the bioreductive activation of nitro­aromatic prodrugs (Wardman *et al.*, 1995 ▶; Fitzsimmons *et al.*, 1996 ▶; Rooseboom *et al.*, 2004 ▶; Chen & Hu, 2009 ▶; Wilson & Hay, 2011 ▶; Wilson *et al.*, 1989 ▶; Denny & Wilson, 1986 ▶; Walton *et al.*, 1989 ▶; Wen *et al.*, 2008 ▶; James *et al.*, 2001 ▶; Patterson *et al.*, 2007 ▶). Our longstanding inter­est in this type of process (Daniels & Gates, 1996 ▶; Junnotula *et al.*, 2009 ▶, 2010 ▶) and our recent inter­est in the bioreductive activation of 6-nitro­quinoline (Rajapakse & Gates, 2012 ▶; Rajapakse *et al.*, 2013 ▶) led us to prepare and characterize the title compound.
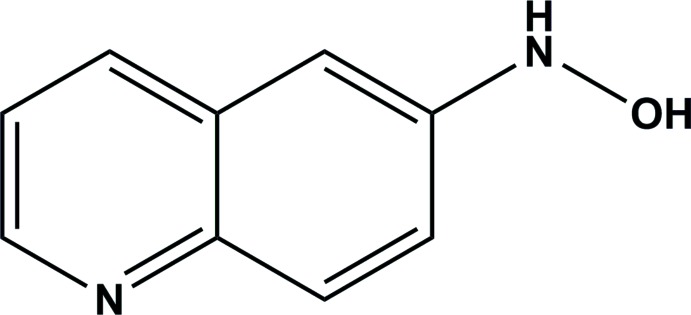


## Structural commentary   

**Figure 1:**
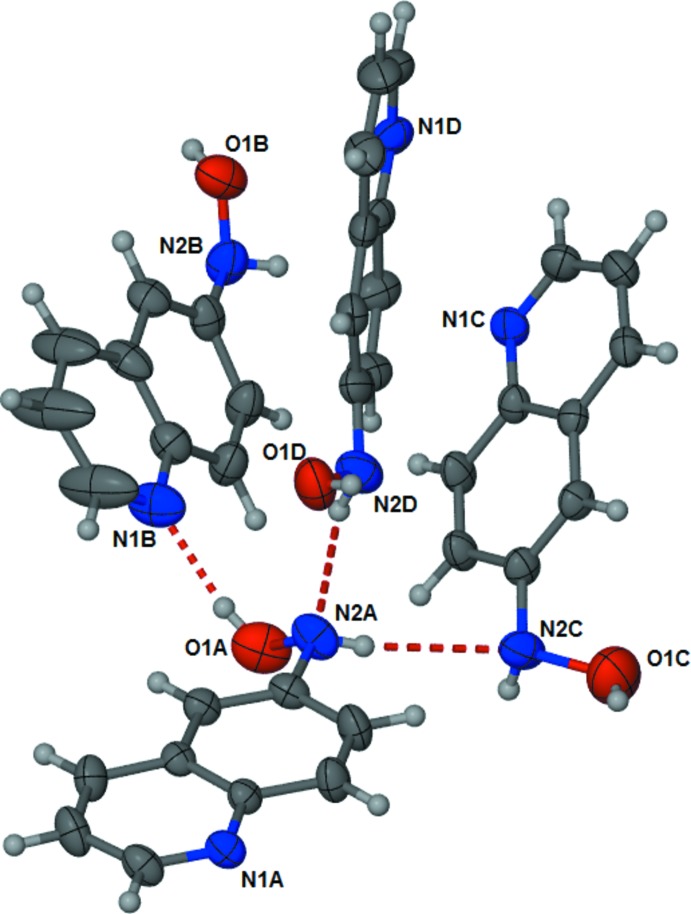
A view of the mol­ecular structure of the four independent mol­ecules (suffixes A, B, C and D) of the title compound, with the atom labelling. Displacement ellipsoids are drawn at the 50% probability level. Hydrogen bonds are shown as dashed lines (see Table 1 ▶ for details).

The title compound, C_9_H_8_N_2_O, crystallized with four independent mol­ecules (*A*, *B*, *C*, and *D*) in the asymmetric unit (Fig. 1 ▶). The O atoms of the hydroxylamino groups in the four independent molecules *A*, *B*, *C*, and *D* are displaced from the aromatic ring planes by 0.745 (5), 0.550 (5), 0.971 (6) and 0.293 (5) Å, respectively. The four mol­ecules are linked *via* one O—H⋯N and two N—H⋯N hydrogen bonds, forming a tetra­mer-like unit (Fig. 1 ▶ and Table 1 ▶).

## Supra­molecular features   

**Figure 2:**
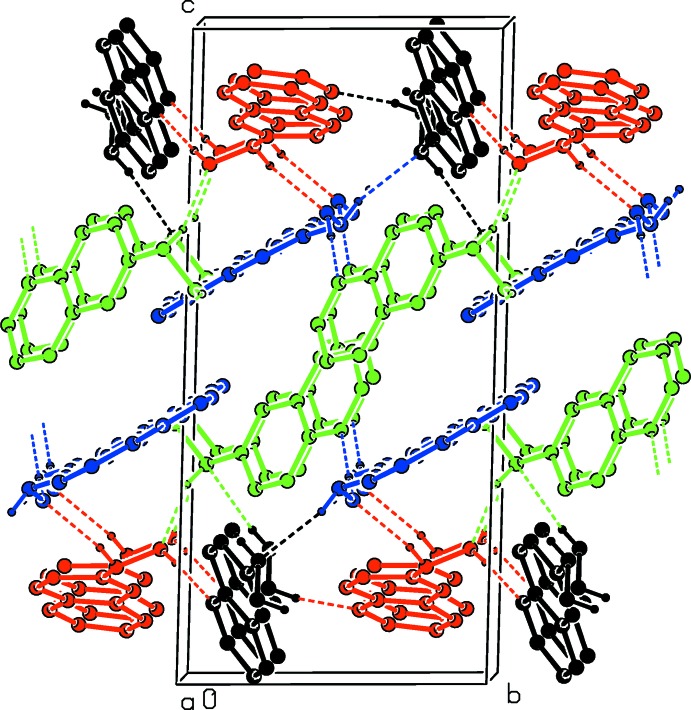
A view along the *a* axis of the crystal packing of the title compound. Hydrogen bonds are shown as dashed lines (see Table 1 ▶ for details). C-bound H atoms have been omitted for clarity. Color key: mol­ecule *A* black, *B* red, *C* green and *D* blue.

In the crystal, the tetra­mer-like units are linked by O—H⋯N and N—H⋯O hydrogen bonds, forming layers parallel to (001); see Table 1 ▶ and Fig. 2 ▶. These layers are linked *via* C—H⋯O hydrogen bonds and a number of C—H⋯π inter­actions (Table 1 ▶), forming a three-dimensional structure.

## Synthesis and crystallization   

To a stirred solution of 6-nitro­quinoline [(1); 0.5 g, 2.87 mmol] in EtOH/CH_2_Cl_2_ (1:1 v/v, 20 ml) at 273 K was added a slurry of Raney nickel (0.5 ml). To this mixture, hydrazine hydrate (10 equivalents) was added dropwise with stirring over the course of 1 h while keeping the solution under an inert atmosphere of nitro­gen gas. The solid was removed by filtration and the resulting solution diluted with water (2 ml) and then extracted with ethyl acetate (2 × 10 ml). The combined organic extracts were washed with brine and dried over sodium sulfate. Column chromatography on silica gel, eluted with ethyl acetate and MeOH/CH_2_Cl_2_, gave the title compound as a yellow solid (yield: 100 mg, 25% yield, *R*_F_ = 0.1 in MeOH/CH_2_Cl_2_ 4:96). It was found to be unstable upon standing in organic solvents. Crystals of the title compound were obtained by dissolving pure product in warm ethyl acetate followed by rapid cooling to give yellow crystals. ^1^H NMR (CD_3_OD, 300 MHz): δ 8.53 (*d*, *J* = 5.0 Hz, 1H), 8.07 (*d*, *J* = 8.0 Hz, 1H), 7.82 (*m*, 1H), 7.33 (*m*, 3H). ^13^C NMR (CD_3_OD, 75.5 MHz) δ 151.40, 147.64, 144.76, 136.72, 131.05, 129.15, 122.54, 121.01, 107.68. HRMS (ESI, *M*+H^+^) *m*/*z* calculated for C_9_H_9_N_2_O: 160.0715; found: 160.0707.

## Refinement   

Crystal data, data collection and structure refinement details are summarized in Table 2 ▶. The NH H atoms were located in a difference Fourier map and freely refined. The OH and C-bound H atoms were included in calculated positions and treated as riding: O—H = 0.84, C—H = 0.95 Å with *U*_iso_(H) = 1.2U_eq_(O,C).

Several crystals examined proved to have multiple domains. The final data crystal, while still a multiple, could be described having primarily two domains and was treated as such. Orientation matrices for the two domains were determined using the program *CELL_NOW* (Bruker, 2008 ▶) and the data were processed further using *TWINABS* (Bruker, 2008 ▶). The model converged well using the HKLF5 data but the final difference map shows several peaks of 0.4 to 0.96 e Å^−3^ near two of the four independent mol­ecules. While this residual electron density could be inter­preted as disorder of parts of those mol­ecules, attempts to model such disorder were unsatis­factory, requiring considerable restraints/constraints to achieve convergence, and were not included in the final model. An alternative explanation of this residual electron density is a possible contribution from crystalline domains not included in the twinning description.

## Supplementary Material

Crystal structure: contains datablock(s) I. DOI: 10.1107/S160053681402193X/su2793sup1.cif

Structure factors: contains datablock(s) I. DOI: 10.1107/S160053681402193X/su2793Isup2.hkl

Supporting information file. DOI: 10.1107/S160053681402193X/su2793Isup3.cml

CCDC reference: 861650

Additional supporting information:  crystallographic information; 3D view; checkCIF report

## Figures and Tables

**Table 1:** Hydrogen-bond geometry (, ) *Cg*1, *Cg*2, *Cg*5, *Cg*8 and *Cg*11 are the centroids of the N1*A*/C1*A*C4*A*/C9*A*, C4*A*C9*A*, C4*B*C9*B*, C4*C*C9*C* and C4*D*C9*D* rings, respectively.

*D*H*A*	*D*H	H*A*	*D* *A*	*D*H*A*
O1*A*H1*OA*N1*B*	0.84	1.88	2.711(5)	170
N2*A*H2*NA*N2*C*	0.78(4)	2.58(4)	3.351(5)	169(4)
N2*D*H2*ND*N2*A*	0.88(4)	2.35(4)	3.204(4)	165(4)
O1*B*H1*OB*N1*A* ^i^	0.84	1.87	2.689(4)	166
O1*C*H1*C*N1*D* ^ii^	0.84	1.82	2.628(5)	160
O1*D*H1*OD*N1*C* ^iii^	0.84	1.93	2.764(4)	172
N2*B*H2*NB*O1*D* ^iv^	0.85(4)	2.14(4)	2.935(4)	157(4)
N2*C*H2*NC*O1*B* ^ii^	0.85(4)	2.12(4)	2.937(4)	159(4)
C7*C*H7*C*O1*B* ^ii^	0.95	2.58	3.300(4)	133
C3*A*H3*A* *Cg*5^v^	0.95	2.64	3.333(3)	130
C3*B*H3*B* *Cg*2^vi^	0.95	2.59	3.265(4)	129
C3*C*H3*C* *Cg*11^vii^	0.95	2.61	3.355(3)	136
C3*D*H3*D* *Cg*8^viii^	0.95	2.85	3.436(4)	121
C7*D*H7*D* *Cg*8	0.95	2.99	3.664(4)	129
C8*B*H8*B* *Cg*1^iv^	0.95	2.85	3.527(4)	129

Symmetry codes: (i) 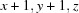; (ii) 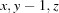; (iii) 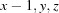; (iv) 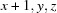; (v) 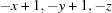; (vi) 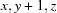; (vii) 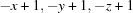; (viii) 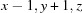.

**Table 2:** Experimental details

Crystal data	
Chemical formula	C_9_H_8_N_2_O
*M*_r_	160.17
Crystal system, space group	Triclinic, *P* 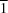
Temperature (K)	173
*a*, *b*, *c* ()	9.3730(15), 9.7117(16), 18.937(3)
, , ()	84.855(2), 83.043(2), 67.477(2)
*V* (^3^)	1578.8(4)
*Z*	8
Radiation type	Mo *K*
(mm^1^)	0.09
Crystal size (mm)	0.35 0.20 0.20

Data collection	
Diffractometer	Bruker APEXII CCD area-detector
Absorption correction	Multi-scan (*TWINABS*; Bruker, 2008 ▶)
*T*_min_, *T*_max_	0.89, 0.98
No. of measured, independent and observed [*I* > 2(*I*)] reflections	31957, 7120, 5311
*R*_int_	0.028
(sin /)_max_ (^1^)	0.650

Refinement	
*R*[*F* ^2^ > 2(*F* ^2^)], *wR*(*F* ^2^), *S*	0.074, 0.224, 1.06
No. of reflections	7120
No. of parameters	454
H-atom treatment	H atoms treated by a mixture of independent and constrained refinement
_max_, _min_ (e ^3^)	0.96, 0.70

Computer programs: *APEX2* and *SAINT* (Bruker, 2008 ▶), *SHELXS97* and *SHELXL97* (Sheldrick, 2008 ▶), *ORTEPIII* (Burnett Johnson, 1996 ▶), *PLATON* (Spek, 2009 ▶) and *publCIF* (Westrip, 2010 ▶).

## supplementary crystallographic information

### Crystal data

**Table:**

C_9_H_8_N_2_O	*Z* = 8
*M**_r_* = 160.17	*F*(000) = 672
Triclinic, *P*1	*D*_x_ = 1.348 Mg m^−^^3^
Hall symbol: -P 1	Mo *K*α radiation, λ = 0.71073 Å
*a* = 9.3730 (15) Å	Cell parameters from 7244 reflections
*b* = 9.7117 (16) Å	θ = 2.4–27.4°
*c* = 18.937 (3) Å	µ = 0.09 mm^−^^1^
α = 84.855 (2)°	*T* = 173 K
β = 83.043 (2)°	Prism, yellow
γ = 67.477 (2)°	0.35 × 0.20 × 0.20 mm
*V* = 1578.8 (4) Å^3^	

### Data collection

**Table:**

Bruker APEXII CCD area-detector diffractometer	7120 independent reflections
Radiation source: fine-focus sealed tube	5311 reflections with *I* > 2σ(*I*)
Graphite monochromator	*R*_int_ = 0.028
ω scans	θ_max_ = 27.5°, θ_min_ = 1.1°
Absorption correction: multi-scan (*TWINABS*; Bruker, 2008)	*h* = −12→12
*T*_min_ = 0.89, *T*_max_ = 0.98	*k* = −12→12
31957 measured reflections	*l* = 0→24

### Refinement

**Table:**

Refinement on *F*^2^	Primary atom site location: structure-invariant direct methods
Least-squares matrix: full	Secondary atom site location: difference Fourier map
*R*[*F*^2^ > 2σ(*F*^2^)] = 0.074	Hydrogen site location: inferred from neighbouring sites
*wR*(*F*^2^) = 0.224	H atoms treated by a mixture of independent and constrained refinement
*S* = 1.06	*w* = 1/[σ^2^(*F*_o_^2^) + (0.1048*P*)^2^ + 1.3081*P*] where *P* = (*F*_o_^2^ + 2*F*_c_^2^)/3
7120 reflections	(Δ/σ)_max_ < 0.001
454 parameters	Δρ_max_ = 0.96 e Å^−^^3^
0 restraints	Δρ_min_ = −0.70 e Å^−^^3^

### Special details

**Table:**

Geometry. All e.s.d.'s (except the e.s.d. in the dihedral angle between two l.s. planes) are estimated using the full covariance matrix. The cell e.s.d.'s are taken into account individually in the estimation of e.s.d.'s in distances, angles and torsion angles; correlations between e.s.d.'s in cell parameters are only used when they are defined by crystal symmetry. An approximate (isotropic) treatment of cell e.s.d.'s is used for estimating e.s.d.'s involving l.s. planes.
Refinement. Refinement of *F*^2^ against ALL reflections. The weighted *R*-factor *wR* and goodness of fit *S* are based on *F*^2^, conventional *R*-factors *R* are based on *F*, with *F* set to zero for negative *F*^2^. The threshold expression of *F*^2^ > σ(*F*^2^) is used only for calculating *R*-factors(gt) *etc*. and is not relevant to the choice of reflections for refinement. *R*-factors based on *F*^2^ are statistically about twice as large as those based on *F*, and *R*-factors based on ALL data will be even larger. The data crystal was a two- domain pseudo-merohedral twin. Data was processed using TWINABS and the final refinement was carried out with the HKLF 5 data. The H atoms on the N2 atoms were located and refined with isotropic thermal parameters. The H atoms on the OH groups appeared in difference maps, but were placed at calculated positions and allowed to find maximum overlap with the electron density in a riding model. Residual electron density near two of the four independent molecules was not amenable to reasonable modelling as disorder and may indicate contribution of an additional minor crystalline domain.

### Fractional atomic coordinates and isotropic or equivalent isotropic displacement parameters (Å^2^)

**Table:**

	*x*	*y*	*z*	*U*_iso_*/*U*_eq_	
O1A	0.5904 (3)	0.2741 (3)	0.13946 (17)	0.0658 (7)	
H1OA	0.5705	0.3626	0.1239	0.079*	
N1A	0.0069 (3)	0.1121 (3)	0.12381 (13)	0.0364 (5)	
C1A	−0.0514 (3)	0.1592 (4)	0.06231 (16)	0.0401 (7)	
H1A	−0.1319	0.1301	0.0515	0.048*	
N2A	0.4790 (3)	0.2709 (4)	0.19701 (17)	0.0485 (7)	
C2A	−0.0015 (3)	0.2493 (4)	0.01207 (16)	0.0450 (7)	
H2A	−0.0468	0.2799	−0.0318	0.054*	
C3A	0.1137 (3)	0.2929 (3)	0.02709 (15)	0.0396 (6)	
H3A	0.1489	0.3547	−0.0063	0.047*	
C4A	0.1803 (3)	0.2457 (3)	0.09231 (14)	0.0291 (5)	
C5A	0.3032 (3)	0.2823 (3)	0.11080 (15)	0.0335 (6)	
H5A	0.3441	0.3418	0.0787	0.040*	
C6A	0.3633 (3)	0.2317 (3)	0.17532 (15)	0.0355 (6)	
C7A	0.3028 (4)	0.1425 (4)	0.22270 (16)	0.0446 (7)	
H7A	0.3433	0.1093	0.2675	0.054*	
C8A	0.1871 (4)	0.1032 (4)	0.20521 (16)	0.0414 (7)	
H8A	0.1498	0.0410	0.2373	0.050*	
C9A	0.1224 (3)	0.1541 (3)	0.13994 (14)	0.0303 (5)	
O1B	0.8871 (3)	0.9329 (3)	0.20442 (14)	0.0576 (6)	
H1OB	0.9352	0.9852	0.1843	0.069*	
N1B	0.5281 (3)	0.5673 (4)	0.1053 (2)	0.0600 (8)	
C1B	0.3984 (5)	0.6582 (5)	0.0806 (3)	0.0928 (19)	
H1B	0.3288	0.6161	0.0685	0.111*	
N2B	0.9495 (3)	0.7941 (3)	0.17819 (17)	0.0483 (7)	
C2B	0.3559 (5)	0.8113 (5)	0.0709 (4)	0.112 (3)	
H2B	0.2598	0.8712	0.0530	0.134*	
C3B	0.4536 (5)	0.8747 (4)	0.0874 (3)	0.0774 (15)	
H3B	0.4278	0.9791	0.0795	0.093*	
C4B	0.5933 (3)	0.7849 (4)	0.11611 (18)	0.0463 (8)	
C5B	0.6977 (3)	0.8430 (4)	0.13731 (17)	0.0416 (7)	
H5B	0.6724	0.9478	0.1354	0.050*	
C6B	0.8354 (3)	0.7477 (4)	0.16065 (16)	0.0405 (7)	
C7B	0.8759 (4)	0.5891 (4)	0.16110 (18)	0.0467 (7)	
H7B	0.9753	0.5234	0.1737	0.056*	
C8B	0.7744 (4)	0.5321 (4)	0.14376 (18)	0.0467 (7)	
H8B	0.8010	0.4271	0.1461	0.056*	
C9B	0.6279 (3)	0.6292 (4)	0.12197 (17)	0.0427 (7)	
O1C	0.6927 (4)	−0.0383 (4)	0.39244 (18)	0.0834 (9)	
H1C	0.6157	−0.0636	0.3975	0.100*	
N1C	0.8919 (3)	0.5060 (3)	0.38596 (13)	0.0395 (6)	
C1C	0.8513 (4)	0.5683 (3)	0.44833 (17)	0.0429 (7)	
H1OC	0.8899	0.6423	0.4563	0.051*	
N2C	0.6800 (4)	0.0668 (3)	0.33013 (15)	0.0482 (7)	
C2C	0.7559 (4)	0.5330 (3)	0.50310 (16)	0.0425 (7)	
H2C	0.7304	0.5817	0.5468	0.051*	
C3C	0.6997 (3)	0.4265 (3)	0.49252 (15)	0.0369 (6)	
H3C	0.6354	0.3999	0.5294	0.044*	
C4C	0.7365 (3)	0.3563 (3)	0.42747 (14)	0.0308 (5)	
C5C	0.6859 (3)	0.2431 (3)	0.41290 (15)	0.0349 (6)	
H5C	0.6239	0.2104	0.4486	0.042*	
C6C	0.7249 (3)	0.1798 (3)	0.34798 (15)	0.0353 (6)	
C7C	0.8151 (4)	0.2316 (3)	0.29446 (15)	0.0386 (6)	
H7C	0.8372	0.1924	0.2484	0.046*	
C8C	0.8702 (4)	0.3363 (3)	0.30816 (15)	0.0394 (6)	
H8C	0.9345	0.3656	0.2723	0.047*	
C9C	0.8333 (3)	0.4017 (3)	0.37463 (14)	0.0325 (6)	
O1D	0.1032 (3)	0.5575 (3)	0.28275 (14)	0.0602 (7)	
H1OD	0.0452	0.5404	0.3169	0.072*	
N1D	0.4155 (3)	0.9499 (3)	0.40248 (15)	0.0443 (6)	
C1D	0.3083 (4)	1.0657 (3)	0.43441 (18)	0.0481 (8)	
H1D	0.3411	1.1335	0.4545	0.058*	
N2D	0.2499 (4)	0.5174 (4)	0.30317 (17)	0.0524 (7)	
C2D	0.1504 (4)	1.0939 (4)	0.4402 (2)	0.0541 (9)	
H2D	0.0783	1.1795	0.4634	0.065*	
C3D	0.1000 (4)	0.9987 (4)	0.41264 (18)	0.0479 (8)	
H3D	−0.0077	1.0171	0.4163	0.058*	
C4D	0.2084 (3)	0.8717 (3)	0.37835 (14)	0.0354 (6)	
C5D	0.1665 (4)	0.7640 (3)	0.35065 (15)	0.0390 (6)	
H5D	0.0602	0.7781	0.3519	0.047*	
C6D	0.2797 (4)	0.6380 (3)	0.32161 (15)	0.0418 (7)	
C7D	0.4385 (4)	0.6209 (4)	0.31648 (18)	0.0468 (8)	
H7D	0.5158	0.5356	0.2952	0.056*	
C8D	0.4805 (4)	0.7250 (3)	0.34159 (17)	0.0443 (7)	
H8D	0.5867	0.7126	0.3373	0.053*	
C9D	0.3671 (3)	0.8519 (3)	0.37414 (15)	0.0363 (6)	
H2NB	1.008 (4)	0.742 (4)	0.209 (2)	0.052 (11)*	
H2NC	0.748 (4)	0.008 (4)	0.301 (2)	0.051 (10)*	
H2NA	0.521 (5)	0.215 (4)	0.227 (2)	0.055 (12)*	
H2ND	0.315 (5)	0.465 (5)	0.269 (2)	0.064 (12)*	

### Atomic displacement parameters (Å^2^)

**Table:**

	*U*^11^	*U*^22^	*U*^33^	*U*^12^	*U*^13^	*U*^23^
O1A	0.0467 (14)	0.0799 (18)	0.080 (2)	−0.0340 (14)	0.0043 (13)	−0.0167 (15)
N1A	0.0344 (12)	0.0440 (13)	0.0362 (13)	−0.0209 (10)	−0.0002 (9)	−0.0056 (10)
C1A	0.0325 (14)	0.0558 (18)	0.0361 (15)	−0.0196 (13)	−0.0028 (11)	−0.0098 (13)
N2A	0.0376 (14)	0.0678 (19)	0.0485 (17)	−0.0266 (14)	−0.0068 (12)	−0.0104 (15)
C2A	0.0372 (15)	0.068 (2)	0.0300 (14)	−0.0185 (14)	−0.0080 (12)	−0.0004 (13)
C3A	0.0355 (14)	0.0502 (17)	0.0299 (14)	−0.0146 (13)	−0.0023 (11)	0.0058 (12)
C4A	0.0251 (12)	0.0322 (12)	0.0276 (12)	−0.0083 (10)	0.0010 (9)	−0.0048 (10)
C5A	0.0308 (13)	0.0392 (14)	0.0319 (14)	−0.0161 (11)	0.0028 (10)	−0.0037 (11)
C6A	0.0304 (13)	0.0407 (15)	0.0367 (15)	−0.0137 (11)	−0.0009 (11)	−0.0098 (11)
C7A	0.0481 (17)	0.0598 (19)	0.0302 (15)	−0.0237 (15)	−0.0111 (12)	0.0017 (13)
C8A	0.0456 (16)	0.0544 (18)	0.0297 (14)	−0.0262 (14)	−0.0048 (12)	0.0050 (12)
C9A	0.0295 (12)	0.0345 (13)	0.0272 (13)	−0.0130 (11)	0.0004 (10)	−0.0031 (10)
O1B	0.0628 (15)	0.0658 (16)	0.0545 (15)	−0.0373 (13)	0.0085 (12)	−0.0137 (12)
N1B	0.0422 (15)	0.0627 (19)	0.080 (2)	−0.0199 (14)	−0.0133 (15)	−0.0185 (16)
C1B	0.046 (2)	0.072 (3)	0.161 (5)	−0.003 (2)	−0.041 (3)	−0.052 (3)
N2B	0.0488 (16)	0.0511 (16)	0.0539 (17)	−0.0289 (13)	−0.0117 (13)	0.0074 (13)
C2B	0.061 (3)	0.069 (3)	0.195 (6)	0.017 (2)	−0.073 (4)	−0.065 (4)
C3B	0.051 (2)	0.047 (2)	0.122 (4)	0.0118 (17)	−0.039 (2)	−0.039 (2)
C4B	0.0311 (14)	0.0577 (19)	0.0453 (17)	−0.0072 (13)	−0.0029 (12)	−0.0232 (15)
C5B	0.0350 (14)	0.0441 (16)	0.0423 (16)	−0.0103 (13)	0.0015 (12)	−0.0127 (13)
C6B	0.0395 (15)	0.0547 (17)	0.0325 (14)	−0.0257 (14)	−0.0020 (11)	0.0058 (12)
C7B	0.0449 (17)	0.0488 (17)	0.0505 (18)	−0.0224 (14)	−0.0153 (14)	0.0120 (14)
C8B	0.0500 (18)	0.0449 (17)	0.0501 (19)	−0.0228 (14)	−0.0152 (14)	0.0095 (14)
C9B	0.0370 (15)	0.0522 (17)	0.0403 (16)	−0.0175 (13)	−0.0037 (12)	−0.0062 (13)
O1C	0.100 (3)	0.088 (2)	0.076 (2)	−0.053 (2)	−0.0081 (18)	0.0077 (17)
N1C	0.0423 (13)	0.0459 (14)	0.0384 (13)	−0.0249 (11)	−0.0076 (10)	−0.0002 (10)
C1C	0.0503 (17)	0.0427 (16)	0.0440 (17)	−0.0245 (14)	−0.0129 (13)	−0.0011 (13)
N2C	0.0646 (18)	0.0509 (16)	0.0415 (15)	−0.0389 (15)	0.0111 (13)	−0.0077 (12)
C2C	0.0516 (17)	0.0421 (16)	0.0336 (15)	−0.0155 (14)	−0.0089 (13)	−0.0044 (12)
C3C	0.0376 (14)	0.0388 (14)	0.0294 (13)	−0.0098 (12)	−0.0031 (11)	0.0017 (11)
C4C	0.0288 (12)	0.0312 (13)	0.0293 (13)	−0.0080 (10)	−0.0057 (10)	0.0039 (10)
C5C	0.0339 (13)	0.0376 (14)	0.0335 (14)	−0.0165 (11)	0.0027 (11)	0.0025 (11)
C6C	0.0366 (14)	0.0394 (14)	0.0340 (14)	−0.0200 (12)	−0.0011 (11)	0.0014 (11)
C7C	0.0469 (16)	0.0486 (16)	0.0280 (13)	−0.0273 (14)	−0.0021 (11)	0.0003 (12)
C8C	0.0457 (16)	0.0526 (17)	0.0284 (14)	−0.0301 (14)	0.0007 (11)	0.0020 (12)
C9C	0.0320 (13)	0.0377 (14)	0.0308 (13)	−0.0162 (11)	−0.0075 (10)	0.0043 (10)
O1D	0.0689 (17)	0.0762 (17)	0.0451 (14)	−0.0371 (14)	−0.0142 (12)	0.0054 (12)
N1D	0.0527 (15)	0.0360 (13)	0.0465 (15)	−0.0218 (12)	0.0028 (12)	0.0010 (11)
C1D	0.063 (2)	0.0353 (15)	0.0463 (18)	−0.0186 (15)	−0.0040 (15)	−0.0015 (13)
N2D	0.0618 (18)	0.0619 (18)	0.0456 (16)	−0.0371 (15)	0.0022 (14)	−0.0095 (14)
C2D	0.059 (2)	0.0370 (16)	0.051 (2)	0.0014 (15)	−0.0083 (16)	−0.0096 (14)
C3D	0.0393 (16)	0.0454 (17)	0.0465 (18)	0.0003 (13)	−0.0104 (13)	−0.0045 (14)
C4D	0.0417 (15)	0.0325 (13)	0.0272 (13)	−0.0090 (11)	−0.0067 (11)	0.0053 (10)
C5D	0.0418 (15)	0.0444 (16)	0.0304 (14)	−0.0158 (13)	−0.0077 (11)	0.0048 (12)
C6D	0.0599 (19)	0.0409 (15)	0.0275 (14)	−0.0243 (14)	0.0018 (12)	−0.0003 (11)
C7D	0.0499 (18)	0.0427 (16)	0.0451 (17)	−0.0187 (14)	0.0152 (14)	−0.0087 (13)
C8D	0.0423 (16)	0.0412 (16)	0.0470 (17)	−0.0173 (13)	0.0113 (13)	−0.0039 (13)
C9D	0.0434 (15)	0.0312 (13)	0.0334 (14)	−0.0161 (12)	0.0028 (11)	0.0038 (11)

### Geometric parameters (Å, º)

**Table:**

O1A—N2A	1.422 (4)	O1C—N2C	1.474 (4)
O1A—H1OA	0.8400	O1C—H1C	0.8400
N1A—C1A	1.316 (4)	N1C—C1C	1.323 (4)
N1A—C9A	1.368 (3)	N1C—C9C	1.366 (4)
C1A—C2A	1.395 (5)	C1C—C2C	1.393 (4)
C1A—H1A	0.9500	C1C—H1OC	0.9500
N2A—C6A	1.393 (4)	N2C—C6C	1.397 (4)
N2A—H2NA	0.78 (4)	N2C—H2NC	0.85 (4)
C2A—C3A	1.369 (4)	C2C—C3C	1.365 (4)
C2A—H2A	0.9500	C2C—H2C	0.9500
C3A—C4A	1.411 (4)	C3C—C4C	1.403 (4)
C3A—H3A	0.9500	C3C—H3C	0.9500
C4A—C9A	1.417 (4)	C4C—C5C	1.412 (4)
C4A—C5A	1.417 (4)	C4C—C9C	1.423 (4)
C5A—C6A	1.378 (4)	C5C—C6C	1.370 (4)
C5A—H5A	0.9500	C5C—H5C	0.9500
C6A—C7A	1.411 (4)	C6C—C7C	1.421 (4)
C7A—C8A	1.363 (4)	C7C—C8C	1.360 (4)
C7A—H7A	0.9500	C7C—H7C	0.9500
C8A—C9A	1.408 (4)	C8C—C9C	1.406 (4)
C8A—H8A	0.9500	C8C—H8C	0.9500
O1B—N2B	1.361 (4)	O1D—N2D	1.372 (4)
O1B—H1OB	0.8400	O1D—H1OD	0.8400
N1B—C1B	1.311 (5)	N1D—C1D	1.322 (4)
N1B—C9B	1.368 (4)	N1D—C9D	1.371 (4)
C1B—C2B	1.385 (7)	C1D—C2D	1.391 (5)
C1B—H1B	0.9500	C1D—H1D	0.9500
N2B—C6B	1.393 (4)	N2D—C6D	1.385 (4)
N2B—H2NB	0.85 (4)	N2D—H2ND	0.88 (4)
C2B—C3B	1.361 (6)	C2D—C3D	1.353 (5)
C2B—H2B	0.9500	C2D—H2D	0.9500
C3B—C4B	1.408 (5)	C3D—C4D	1.414 (4)
C3B—H3B	0.9500	C3D—H3D	0.9500
C4B—C5B	1.413 (4)	C4D—C5D	1.410 (4)
C4B—C9B	1.417 (5)	C4D—C9D	1.418 (4)
C5B—C6B	1.366 (4)	C5D—C6D	1.382 (4)
C5B—H5B	0.9500	C5D—H5D	0.9500
C6B—C7B	1.438 (5)	C6D—C7D	1.426 (5)
C7B—C8B	1.353 (4)	C7D—C8D	1.356 (5)
C7B—H7B	0.9500	C7D—H7D	0.9500
C8B—C9B	1.422 (4)	C8D—C9D	1.415 (4)
C8B—H8B	0.9500	C8D—H8D	0.9500
			
N2A—O1A—H1OA	109.5	N2C—O1C—H1C	109.5
C1A—N1A—C9A	118.5 (2)	C1C—N1C—C9C	117.2 (3)
N1A—C1A—C2A	123.7 (3)	N1C—C1C—C2C	124.5 (3)
N1A—C1A—H1A	118.2	N1C—C1C—H1OC	117.7
C2A—C1A—H1A	118.2	C2C—C1C—H1OC	117.7
C6A—N2A—O1A	111.7 (3)	C6C—N2C—O1C	108.0 (3)
C6A—N2A—H2NA	110 (3)	C6C—N2C—H2NC	112 (3)
O1A—N2A—H2NA	109 (3)	O1C—N2C—H2NC	99 (3)
C3A—C2A—C1A	118.8 (3)	C3C—C2C—C1C	118.5 (3)
C3A—C2A—H2A	120.6	C3C—C2C—H2C	120.8
C1A—C2A—H2A	120.6	C1C—C2C—H2C	120.8
C2A—C3A—C4A	119.9 (3)	C2C—C3C—C4C	120.3 (3)
C2A—C3A—H3A	120.1	C2C—C3C—H3C	119.8
C4A—C3A—H3A	120.1	C4C—C3C—H3C	119.8
C3A—C4A—C9A	117.3 (2)	C3C—C4C—C5C	123.8 (3)
C3A—C4A—C5A	123.2 (3)	C3C—C4C—C9C	116.8 (3)
C9A—C4A—C5A	119.5 (2)	C5C—C4C—C9C	119.4 (2)
C6A—C5A—C4A	120.1 (3)	C6C—C5C—C4C	120.9 (2)
C6A—C5A—H5A	119.9	C6C—C5C—H5C	119.6
C4A—C5A—H5A	119.9	C4C—C5C—H5C	119.6
C5A—C6A—N2A	122.0 (3)	C5C—C6C—N2C	123.9 (3)
C5A—C6A—C7A	119.7 (3)	C5C—C6C—C7C	119.1 (3)
N2A—C6A—C7A	118.2 (3)	N2C—C6C—C7C	116.9 (3)
C8A—C7A—C6A	121.0 (3)	C8C—C7C—C6C	121.0 (3)
C8A—C7A—H7A	119.5	C8C—C7C—H7C	119.5
C6A—C7A—H7A	119.5	C6C—C7C—H7C	119.5
C7A—C8A—C9A	120.6 (3)	C7C—C8C—C9C	120.9 (3)
C7A—C8A—H8A	119.7	C7C—C8C—H8C	119.5
C9A—C8A—H8A	119.7	C9C—C8C—H8C	119.5
N1A—C9A—C8A	119.2 (2)	N1C—C9C—C8C	118.8 (2)
N1A—C9A—C4A	121.8 (2)	N1C—C9C—C4C	122.7 (2)
C8A—C9A—C4A	119.0 (2)	C8C—C9C—C4C	118.6 (3)
N2B—O1B—H1OB	109.5	N2D—O1D—H1OD	109.5
C1B—N1B—C9B	117.1 (3)	C1D—N1D—C9D	117.7 (3)
N1B—C1B—C2B	124.5 (4)	N1D—C1D—C2D	123.8 (3)
N1B—C1B—H1B	117.7	N1D—C1D—H1D	118.1
C2B—C1B—H1B	117.7	C2D—C1D—H1D	118.1
O1B—N2B—C6B	111.6 (3)	O1D—N2D—C6D	112.9 (3)
O1B—N2B—H2NB	104 (3)	O1D—N2D—H2ND	107 (3)
C6B—N2B—H2NB	118 (3)	C6D—N2D—H2ND	115 (3)
C3B—C2B—C1B	119.1 (4)	C3D—C2D—C1D	119.6 (3)
C3B—C2B—H2B	120.4	C3D—C2D—H2D	120.2
C1B—C2B—H2B	120.4	C1D—C2D—H2D	120.2
C2B—C3B—C4B	119.8 (4)	C2D—C3D—C4D	119.6 (3)
C2B—C3B—H3B	120.1	C2D—C3D—H3D	120.2
C4B—C3B—H3B	120.1	C4D—C3D—H3D	120.2
C3B—C4B—C5B	123.2 (3)	C5D—C4D—C3D	123.3 (3)
C3B—C4B—C9B	116.6 (3)	C5D—C4D—C9D	119.5 (3)
C5B—C4B—C9B	120.3 (3)	C3D—C4D—C9D	117.2 (3)
C6B—C5B—C4B	119.6 (3)	C6D—C5D—C4D	120.1 (3)
C6B—C5B—H5B	120.2	C6D—C5D—H5D	119.9
C4B—C5B—H5B	120.2	C4D—C5D—H5D	119.9
C5B—C6B—N2B	123.6 (3)	C5D—C6D—N2D	123.4 (3)
C5B—C6B—C7B	120.0 (3)	C5D—C6D—C7D	119.8 (3)
N2B—C6B—C7B	116.1 (3)	N2D—C6D—C7D	116.5 (3)
C8B—C7B—C6B	120.9 (3)	C8D—C7D—C6D	120.8 (3)
C8B—C7B—H7B	119.6	C8D—C7D—H7D	119.6
C6B—C7B—H7B	119.6	C6D—C7D—H7D	119.6
C7B—C8B—C9B	120.0 (3)	C7D—C8D—C9D	120.4 (3)
C7B—C8B—H8B	120.0	C7D—C8D—H8D	119.8
C9B—C8B—H8B	120.0	C9D—C8D—H8D	119.8
N1B—C9B—C4B	122.8 (3)	N1D—C9D—C8D	118.5 (3)
N1B—C9B—C8B	118.2 (3)	N1D—C9D—C4D	122.1 (3)
C4B—C9B—C8B	118.9 (3)	C8D—C9D—C4D	119.4 (3)
			
C9A—N1A—C1A—C2A	0.4 (4)	C9C—N1C—C1C—C2C	1.1 (5)
N1A—C1A—C2A—C3A	−0.4 (5)	N1C—C1C—C2C—C3C	0.2 (5)
C1A—C2A—C3A—C4A	0.4 (5)	C1C—C2C—C3C—C4C	−0.7 (4)
C2A—C3A—C4A—C9A	−0.2 (4)	C2C—C3C—C4C—C5C	178.6 (3)
C2A—C3A—C4A—C5A	178.0 (3)	C2C—C3C—C4C—C9C	−0.1 (4)
C3A—C4A—C5A—C6A	−179.6 (3)	C3C—C4C—C5C—C6C	179.5 (3)
C9A—C4A—C5A—C6A	−1.4 (4)	C9C—C4C—C5C—C6C	−1.9 (4)
C4A—C5A—C6A—N2A	−176.9 (3)	C4C—C5C—C6C—N2C	179.5 (3)
C4A—C5A—C6A—C7A	0.4 (4)	C4C—C5C—C6C—C7C	−1.3 (4)
O1A—N2A—C6A—C5A	−39.0 (4)	O1C—N2C—C6C—C5C	−40.2 (4)
O1A—N2A—C6A—C7A	143.7 (3)	O1C—N2C—C6C—C7C	140.5 (3)
C5A—C6A—C7A—C8A	1.1 (5)	C5C—C6C—C7C—C8C	3.8 (5)
N2A—C6A—C7A—C8A	178.5 (3)	N2C—C6C—C7C—C8C	−176.9 (3)
C6A—C7A—C8A—C9A	−1.6 (5)	C6C—C7C—C8C—C9C	−3.1 (5)
C1A—N1A—C9A—C8A	−179.3 (3)	C1C—N1C—C9C—C8C	178.1 (3)
C1A—N1A—C9A—C4A	−0.2 (4)	C1C—N1C—C9C—C4C	−2.0 (4)
C7A—C8A—C9A—N1A	179.7 (3)	C7C—C8C—C9C—N1C	179.8 (3)
C7A—C8A—C9A—C4A	0.6 (4)	C7C—C8C—C9C—C4C	−0.1 (4)
C3A—C4A—C9A—N1A	0.2 (4)	C3C—C4C—C9C—N1C	1.5 (4)
C5A—C4A—C9A—N1A	−178.2 (2)	C5C—C4C—C9C—N1C	−177.3 (2)
C3A—C4A—C9A—C8A	179.2 (3)	C3C—C4C—C9C—C8C	−178.7 (2)
C5A—C4A—C9A—C8A	0.9 (4)	C5C—C4C—C9C—C8C	2.6 (4)
C9B—N1B—C1B—C2B	−0.5 (9)	C9D—N1D—C1D—C2D	−0.6 (5)
N1B—C1B—C2B—C3B	0.4 (11)	N1D—C1D—C2D—C3D	0.6 (5)
C1B—C2B—C3B—C4B	−2.1 (9)	C1D—C2D—C3D—C4D	0.0 (5)
C2B—C3B—C4B—C5B	−177.3 (5)	C2D—C3D—C4D—C5D	177.5 (3)
C2B—C3B—C4B—C9B	3.9 (7)	C2D—C3D—C4D—C9D	−0.6 (4)
C3B—C4B—C5B—C6B	−176.0 (4)	C3D—C4D—C5D—C6D	−176.1 (3)
C9B—C4B—C5B—C6B	2.8 (5)	C9D—C4D—C5D—C6D	2.0 (4)
C4B—C5B—C6B—N2B	175.7 (3)	C4D—C5D—C6D—N2D	170.0 (3)
C4B—C5B—C6B—C7B	2.3 (5)	C4D—C5D—C6D—C7D	−3.2 (4)
O1B—N2B—C6B—C5B	31.3 (4)	O1D—N2D—C6D—C5D	30.3 (4)
O1B—N2B—C6B—C7B	−155.1 (3)	O1D—N2D—C6D—C7D	−156.3 (3)
C5B—C6B—C7B—C8B	−5.0 (5)	C5D—C6D—C7D—C8D	1.9 (5)
N2B—C6B—C7B—C8B	−178.9 (3)	N2D—C6D—C7D—C8D	−171.8 (3)
C6B—C7B—C8B—C9B	2.6 (5)	C6D—C7D—C8D—C9D	0.7 (5)
C1B—N1B—C9B—C4B	2.5 (6)	C1D—N1D—C9D—C8D	−178.2 (3)
C1B—N1B—C9B—C8B	−175.3 (4)	C1D—N1D—C9D—C4D	−0.1 (4)
C3B—C4B—C9B—N1B	−4.2 (5)	C7D—C8D—C9D—N1D	176.3 (3)
C5B—C4B—C9B—N1B	176.9 (3)	C7D—C8D—C9D—C4D	−1.9 (4)
C3B—C4B—C9B—C8B	173.7 (4)	C5D—C4D—C9D—N1D	−177.5 (3)
C5B—C4B—C9B—C8B	−5.2 (5)	C3D—C4D—C9D—N1D	0.7 (4)
C7B—C8B—C9B—N1B	−179.6 (3)	C5D—C4D—C9D—C8D	0.6 (4)
C7B—C8B—C9B—C4B	2.5 (5)	C3D—C4D—C9D—C8D	178.8 (3)

### Hydrogen-bond geometry (Å, º)

Cg1, Cg2, Cg5, Cg8 and Cg11 are the centroids of the N1A/C1A–C4A/C9A, C4A–C9A, C4B–C9B, C4C–C9C and C4D–C9D rings, respectively.

**Table:**

*D*—H···*A*	*D*—H	H···*A*	*D*···*A*	*D*—H···*A*
O1*A*—H1*OA*···N1*B*	0.84	1.88	2.711 (5)	170
N2*A*—H2*NA*···N2*C*	0.78 (4)	2.58 (4)	3.351 (5)	169 (4)
N2*D*—H2*ND*···N2*A*	0.88 (4)	2.35 (4)	3.204 (4)	165 (4)
O1*B*—H1*OB*···N1*A*^i^	0.84	1.87	2.689 (4)	166
O1*C*—H1*C*···N1*D*^ii^	0.84	1.82	2.628 (5)	160
O1*D*—H1*OD*···N1*C*^iii^	0.84	1.93	2.764 (4)	172
N2*B*—H2*NB*···O1*D*^iv^	0.85 (4)	2.14 (4)	2.935 (4)	157 (4)
N2*C*—H2*NC*···O1*B*^ii^	0.85 (4)	2.12 (4)	2.937 (4)	159 (4)
C7*C*—H7*C*···O1*B*^ii^	0.95	2.58	3.300 (4)	133
C3*A*—H3*A*···*Cg*5^v^	0.95	2.64	3.333 (3)	130
C3*B*—H3*B*···*Cg*2^vi^	0.95	2.59	3.265 (4)	129
C3*C*—H3*C*···*Cg*11^vii^	0.95	2.61	3.355 (3)	136
C3*D*—H3*D*···*Cg*8^viii^	0.95	2.85	3.436 (4)	121
C7*D*—H7*D*···*Cg*8	0.95	2.99	3.664 (4)	129
C8*B*—H8*B*···*Cg*1^iv^	0.95	2.85	3.527 (4)	129

Symmetry codes: (i) *x*+1, *y*+1, *z*; (ii) *x*, *y*−1, *z*; (iii) *x*−1, *y*, *z*; (iv) *x*+1, *y*, *z*; (v) −*x*+1, −*y*+1, −*z*; (vi) *x*, *y*+1, *z*; (vii) −*x*+1, −*y*+1, −*z*+1; (viii) *x*−1, *y*+1, *z*.
